# Family-Based Interventions for Pediatric Obesity: A Comprehensive Systematic Review and Meta-Analysis of Their Effectiveness

**DOI:** 10.7759/cureus.65919

**Published:** 2024-08-01

**Authors:** Hector I Guerra Toro, Arturo P Jaramillo, Valeria M Caceres

**Affiliations:** 1 General Practice, Pontificia Universidad Catolica del Ecuador, Quito, ECU; 2 General Practice, Universidad Estatal de Guayaquil, Machala, ECU; 3 General Practice, Universidad de Las Américas, Quito, ECU

**Keywords:** pediatrics education, family-based treatment, children obesity, physical activity behaviour, nutrition and metabolism .obesity. dietary fiber

## Abstract

Genetics can influence obesity, and when it affects both parents and children, there is a high risk of developing cardiometabolic diseases. Studies have indicated that family-based treatment (FBT) is a cost-effective and successful option for achieving significant weight changes in both children and parents. While specialized clinics offer FBT, primary care settings, where most pediatric care takes place, may not have the necessary resources or expertise to provide intensive behavioral interventions for childhood obesity. Based on early findings, FBT could potentially have a positive impact on siblings as well, as when treated children and parents experience behavioral changes, it can also have a beneficial effect on their untreated siblings. Parents play a crucial role in shaping their children's behavior, and siblings often have a stronger influence on them than their parents or friends.

For our meta-analysis, we utilized three graphical models created using RevMan 5.4, based on the selected articles. To develop our systematic review, we thoroughly analyzed a total of 10 articles. The subgroup analysis within these studies assessed the effectiveness of FBT for overweight children, revealing no significant differences between groups (p=0.77). This suggests that based on their BMI, FBT may not have a statistically significant impact on weight loss in overweight children. However, each study reviewed showed statistical significance (p<0.05). The findings of our meta-analysis underscore the need for more robust evidence and larger randomized controlled trials (RCTs) to enhance our understanding of FBT's benefits in pediatric obesity. This will be crucial for reducing the rising prevalence of obesity and maintaining lower incidence rates.

## Introduction and background

In the United States, the rate of childhood obesity has significantly increased over the last three decades. Currently, more than a third of preadolescent children are considered overweight or obese [1]. The rise in childhood obesity has a significant impact on the likelihood of developing severe obesity in adulthood. This, in turn, increases the chances of experiencing chronic diseases and disabilities later in life [2,3]. The American Academy of Pediatrics (AAP) suggests a step-by-step approach to addressing pediatric overweight and obesity, starting with Stage 1, Prevention Plus. This stage promotes the adoption of healthier lifestyle choices to improve the BMI [4]. Nevertheless, numerous pediatric practices face challenges in implementing these guidelines due to time constraints and limited access to weight loss experts for patient referrals. Thus, it is crucial to have weight-management treatments that are both effective and easily accessible in pediatric primary care settings. Primary care-based interventions, like Let's Go 5-2-1-0, have been proven to boost provider confidence in addressing weight concerns and providing guidance on healthy habits. These interventions involve training providers and staff to promote healthy habits and utilize screening tools for diet and activity [5,6]. Although these interventions have proven to be effective, they are not commonly embraced due to time constraints and doubts about the effectiveness of counseling in modifying behaviors in parents and children [7]. Due to limited resources, most pediatric patients with overweight and obesity are not referred to registered dietitians. A survey of 600 pediatric care providers found that a majority of them did not have access to a pediatric dietitian and lacked referral sources for pediatric weight management [8,9]. Given the time constraints that pediatricians face when it comes to weight counseling and the limited availability of referral resources, AAP has recognized the importance of referral centers in a comprehensive pediatric obesity treatment plan [10]. These centers offer centralized resources for families, providing counseling on nutrition, activity, and weight management [11]. Unfortunately, several families encounter challenges when trying to make use of these resources, including issues with distance, transportation, expenses, and coordinating family schedules. These obstacles are especially noticeable for children from low-income families, who have limited access to care and are at the greatest risk of obesity [12]. To fill this void, a cost-effective and readily available resource that focuses on parents and is accessible via telephone could greatly decrease the prevalence of childhood obesity in clinical settings. Parent coaching in pediatric weight management is effective, sometimes even more so than interventions involving both parents and children. Additionally, these interventions tend to be more cost-effective [13]. Studies have indicated that telephone coaching can be just as effective as in-person visits in maintaining childhood weight loss. However, there is limited research on the primary reductions in BMI. A study discovered that regular phone coaching sessions over a year had a positive impact on childhood BMI for families recruited from pediatric practices [13]. Nevertheless, it is still uncertain whether more frequent sessions, like weekly phone coaching, are more effective over a shorter period [13]. The Fitline pediatric practice-based referral program offers a compact provider intervention, followed by a referral to eight weekly coaching calls with a nutritionist. The program aims to assist families in implementing lifestyle changes that are recommended by AAP. This program offers a convenient referral resource for providers, allowing them to access trained nutritionists who can guide parents on improving their child's weight-related behaviors. A previous study where the Fitline program was tested without randomization and included a control group showed significant reductions in BMI and improvements in weight-related behaviors [13]. In this study, 40 parents and their children aged 8-12 years with a BMI ≥85 percentile were recruited from an academic pediatric practice serving a diverse population, achieving full retention. The children, who had an average age of 9.6 years [standard deviation (SD): 1.4] and a mean BMI of 27.2 (SD: 3.4), came from predominantly low-income families (insured by MassHealth/Medicaid) and had diverse racial and ethnic backgrounds: 25% Hispanic, 12.5% Black, 47.5% White, and 15% Multiracial.

The Fitline group demonstrated a decrease in mean BMI of -0.45 BMI units (SD: 0.99; t-test: -2.86, p=0.007) from the start of the study till the three-month follow-up. In contrast, the control group experienced an average increase in BMI of 0.35 BMI units (SD: 0.96; t-test: 2.42, p=0.02). The BMI change between the two groups showed a significant difference (0.85, t-test: 3.59, p<0.0006), resulting in an approximate 8-pound variation over three months. Notable enhancements were also noted in dietary and sedentary habits, such as decreased intake of fast food, desserts, fruit juice, and sweetened beverages, as well as reduced reliance on computers and video games. In addition, there was a notable rise in the consumption of fruits and vegetables (p=0.002) and a tendency towards engaging in physical activity for at least 60 minutes on more days [13]. The main objective of this systematic review and meta-analysis is to thoroughly analyze the most recent studies on the efficacy of family-based treatment (FBT) in tackling pediatric obesity. We aim to offer a thorough assessment of these interventions to enhance clinical understanding and improve treatment outcomes for pediatric obesity.

## Review

Methods

Review Protocol and Search for Studies

For this systematic review, we followed the protocols outlined in the Preferred Reporting Items for Systematic Reviews and Meta-Analyses (PRISMA) [14]. The process for selecting articles was carried out by independent researchers who performed extensive searches on databases such as PubMed, PubMed Central, and the Cochrane Library. The specific search strategies used are detailed in Table 1.

**Table 1:** Search strategy

Search strategy - keywords	Databases used	Number of papers identified
Pediatric Obesity AND Physical Activity and Family-Base Program	Pubmed	539
("Pediatric Obesity/complications"[Majr] OR "Pediatric Obesity/diet therapy"[Majr] OR "Pediatric Obesity/prevention and control"[Majr] OR "Pediatric Obesity/therapy"[Majr])	Pubmed Central (PMC)	2438
"Pediatric Obesity [tw]" AND " Family-Base Program [tiab]" AND "Physical activity [all]"	Cochrane Library	1130

Inclusion and Exclusion Criteria

Two independent authors utilized the Covidence software to screen the search results obtained from two databases per pre-established inclusion and exclusion criteria (Table 2).

**Table 2:** Inclusion and exclusion criteria

Inclusion criteria	Exclusion criteria
Free, full text about the related subject	Articles that include pharmaceutical trials
Articles from the past 10 years	Articles from 2013 and before
English-language articles	Non-English studies
Prospective or retrospective studies	Case reports, systematic reviews, or literature reviews
Human trials	Animal trials

Data Collection Process

Throughout our meticulous review of the existing literature on family-based interventions in pediatric obesity, we uncovered several key observations. The primary focus of the studies we examined revolved around the structural design of the interventions and the outcomes reported in each article.

Risk of Bias Evaluation

To assess the integrity and reliability of the selected studies, we employed the Cochrane risk of bias tool, a recognized standard for evaluating randomized controlled trials (RCTs). This tool is widely acclaimed for its effectiveness in analyzing the quality of case-series studies [15]. Our team conducted a thorough, unbiased review of each study, and any disagreements among the reviewers were resolved through detailed discussions, ensuring a comprehensive and fair assessment of potential biases.

Statistical Methodology

We utilized Review Manager (RevMan) version 5.4 (2020), developed by The Cochrane Collaboration at The Nordic Cochrane Centre, Copenhagen, Denmark, for all statistical analyses. The outcomes of the studies were articulated using mean differences, supplemented by 95% confidence intervals (CI). For the aggregation of data across studies, an odds ratio (OR) effects model was employed. In instances where standard deviations or standard errors were not reported within the studies, we applied the statistical methods outlined by Mantel-Haenszel et al. Given the varied designs and demographic characteristics of the included studies, a fixed-effect model was preferred over a random-effect model due to its suitability for managing the expected high variance.

Forest plots played a crucial role in visually summarizing the combined results, while the chi-square test was pivotal in detecting any subgroup disparities. The extent of heterogeneity among the studies was quantified using Higgins I^2^ statistic. To assess publication bias, funnel plots were scrutinized visually, with a significance cut-off set at p<0.05.

Research Outcomes and Study Selection

**Figure 1:**
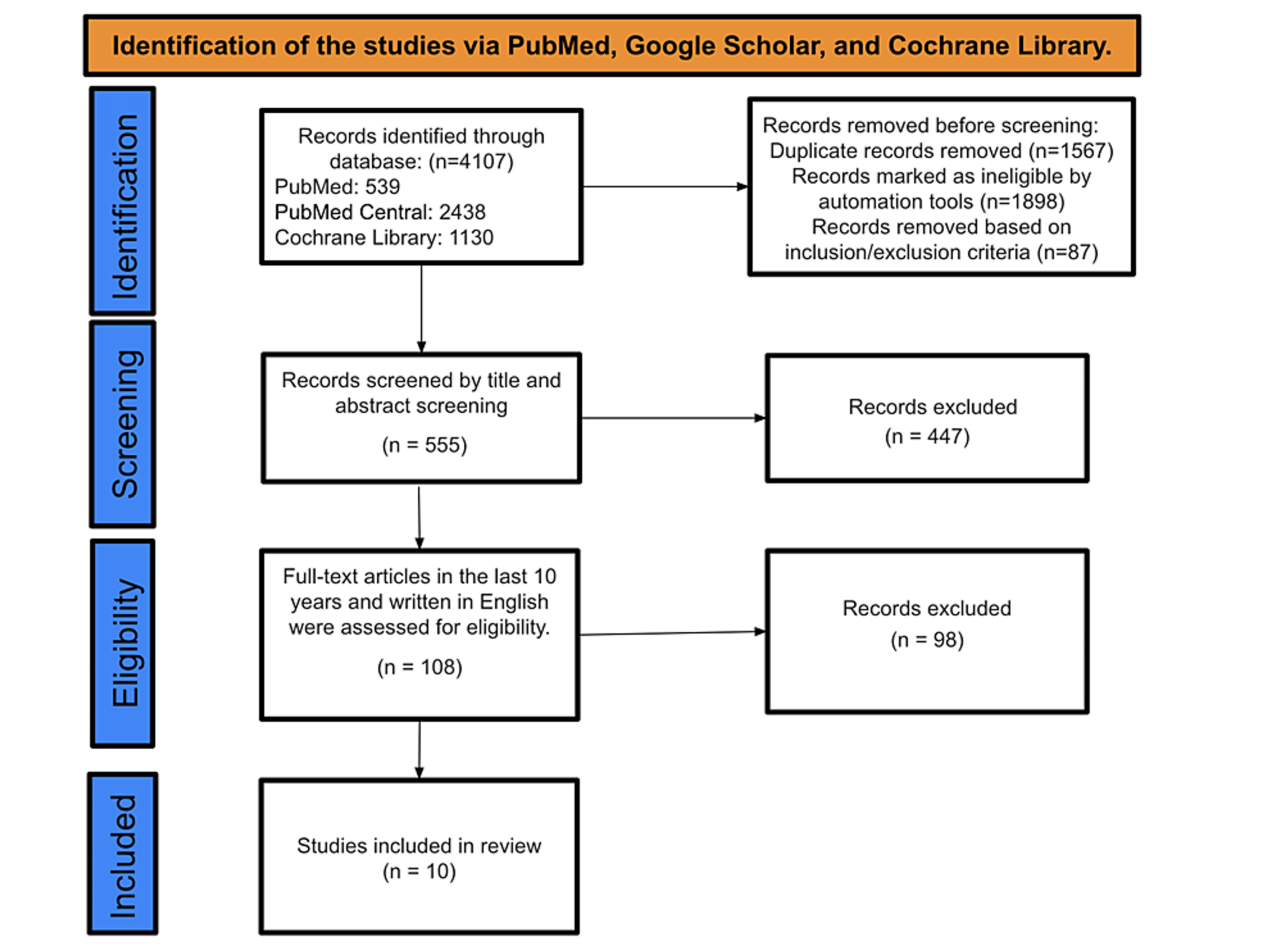
PRISMA diagram depicting the selection of studies PRISMA: Preferred Reporting Items for Systematic Reviews and Meta-Analyses

Our initial search across multiple databases, including PubMed, Cochrane Library, and PMC, yielded a total of 4,107 studies. After applying inclusion and exclusion criteria, 87 studies were deemed ineligible, and automation tools identified an additional 1,898 articles as irrelevant. We also eliminated 1,567 duplicates during our review process. The screening of titles and abstracts led to the further exclusion of 447 studies that did not align with our research objectives. After a detailed examination based on the availability of full texts in English and publication within the last decade, 98 out of the 108 initially screened studies were discarded. Ultimately, only 10 studies met all criteria and were included in our final data analysis as depicted in Figure 1, which illustrates the identification and selection of studies via various databases and registers.

Table 3 presents an in-depth description of the articles we decided to use.

**Table 3:** Summary of studies included BMI: body mass index; BMI-SDS: BMI standard deviation score; BMIz: BMI z-score; FBT: family-based treatment; FBT-PO: FBT for pediatric obesity; HHHK-Preschool: The Healthy Homes/Healthy Kids Preschool; LAUNCH: home- and clinic-based behavioral intervention; NEC: nutrition education counseling; PA: physical activity; RCT: randomized controlled trial; STC: standard care

Author	Year of publication	Study design	Primary research	Outcome evaluation
Stark et al. [11]	2019	RCT	Children between the ages of 2 and 5 years above the 95th percentile for BMI or age and sex were included in the study. They were recruited from 27 pediatrician offices across 10 recruitment cycles between March 12, 2012, and June 8, 2015. Follow-ups were conducted 6 and 12 months after treatment	LAUNCH showed a significant decrease in child BMI% 50th compared to MI at the 12-month follow-up, and compared to STC at the 6-month follow-up. The effect sizes remained consistent when compared to STC at the 12-month follow-up
Pbert et al. [13]	2021	RCT	494 parents and their children ages 8–12 who have a BMI of ≥85th percentile are currently being recruited. The main focus is on the child's BMI, with additional attention given to their diet and PA at different time points	Providing telephonic coaching to parents to enhance their child's diet and PA is a cutting-edge approach with the potential to make a substantial impact on public health. This evidence-based model can be easily implemented in pediatric practices
Epstein et al. [16]	2023	RCT	In this RCT conducted in four different US settings, a total of 452 children between the ages of 6 and 12 years who were overweight or obese, along with their parents and 106 siblings, were enrolled. Participants were assigned to undergo FBT or usual care and were followed up for 24 months. The trial took place from November 2017 to August 2021	In this study with a follow-up period of 24 months, children, parents, and siblings who were overweight or obese and participated in FBT achieved significantly better weight loss results compared to those who received usual care. The outcomes were measured by the percentage above the median body mass index for their age and sex
Kinlin et al. [17]	2022	RCT	During the period from 2017 to 2020, a comprehensive study was carried out in the obesity management clinic of a prominent children's hospital in Toronto, Canada, utilizing various research methods	During a period of 21 months, a total of 11 parent-child dyads were selected for the study. Out of the 6 dyads assigned to the intervention group, unfortunately, 3 did not actively engage in the group sessions or home visits
Loeb et al. [18]	2019	RCT	A total of 77 adolescents were assigned to either FBT-PO or NEC treatment at two different locations.	BT-PO, although it may not result in a significant decrease in BMI z-score, has the potential to prevent further weight gain in these adolescents
Ojeda-Rodríguez et al. [19]	2018	RCT	An RCT was conducted with 107 participants who were divided into two groups: one receiving usual care and the other receiving intensive care, which included following a moderate hypocaloric Mediterranean diet and receiving NEC	Both groups successfully achieved a noteworthy decrease in BMI-SDS, glucose, and total cholesterol levels
Rohde et al. [20]	2017	RCT	Out of a group of 635 Danish preschool children, 285 successfully participated in the intervention and provided complete information on their dietary intake. This group consisted of children who had a high birth weight, a high maternal pre-pregnancy BMI, or a low maternal educational level	During the 15-month intervention, the children in the intervention group consumed less energy compared to the control group
Boutelle et al. [21]	2017	RCT	An RCT 2-arm non-inferiority trial was conducted at the University of California, San Diego, an academic medical center, from July 2011 to July 2015	Treatment involving parents proved to be just as effective in promoting weight loss in children, along with several other positive outcomes
Stettler et al. [22]	2015	RCT	An efficacy trial was conducted using cluster randomization at the practice level. The trial involved a 12-session, 12-month intervention aimed at reducing sweetened beverage consumption, as well as a comprehensive dietary and PA intervention. These interventions were compared to a control intervention among children aged 8-12 years	By engaging families in an obesity prevention program, pediatric primary care clinicians, who receive compensation, training, and ongoing support from behavioral specialists, can have a positive effect on children's BMI.
Sherwood et al. [23]	2015	RCT	A total of 60 parent-children were selected from pediatric primary care clinics and divided into two groups. The first group participated in the Busy Bodies/Better Bites Obesity Prevention Arm, while the second group was part of the Healthy Tots/Safe Spots safety/injury prevention Contact Control Arm	The results of a pilot study in preschool children show that a pediatric primary care-based obesity prevention intervention, which includes brief counseling from healthcare providers and phone coaching for parents, is both feasible and acceptable. The study also suggests that this intervention has the potential to be effective in children who are already overweight.

Table 4 presents the Cochrane risk of bias tool analysis for randomized controlled trials, as it pertains to the studies included in this review.

**Table 4:** Cochrane risk of bias tool +: The article does not have any of the biases described in the table. -: Article bias is presented. ?: The article bias was not found in the study

Studies	Random sequence generation (selection bias)	Allocation concealment (selection bias)	Blinding of participants	Blinding of personnel/care providers (performance bias)	Blinding of outcome assessor (detection bias)	Incomplete outcome data (attrition bias)	Selective reporting (reporting bias)	Other biases	Overall
Stark et al. [11]	+	+	+	+	+	+	+	-	7/8
Pbert et al. [13]	+	+	+	+	+	+	+	-	7/8
Epstein et al. [16]	+	+	+	+	?	+	+	-	6/8
Kinlin et al. [17]	+	+	+	+	+	+	+	-	7/8
Loeb et al. [18]	+	+	+	+	+	+	+	-	7/8
Ojeda-Rodríguez et al. [19]	+	+	+	+	+	+	+	-	7/8
Rohde et al. [20]	+	+	+	+	?	+	+	-	6/8
Boutelle et al. [21]	+	+	+	+	-	+	+	-	7/8
Stettler et al. [22]	+	+	+	+	?	+	+	-	6/8
Sherwood et al. [23]	+	+	+	+	?	+	+	-

Meta-analysis of outcomes

**Figure 2:**
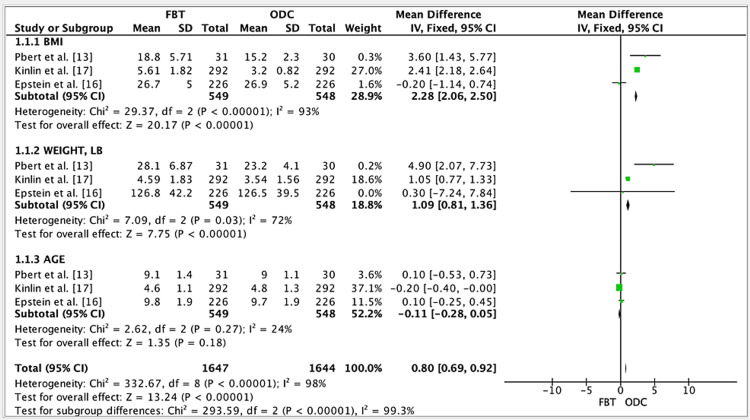
Forest plot to assess FBT vs. ODC* *Created using the mean and standard deviation (SD) of three studies. References: [13,16-17] BMI: body mass index; CI: confidence interval; FBT: family-based treatment; ODC: occasional diet counseling

The results of three studies showed a mean difference of 0.80 for the efficacy of FBT vs. occasional diet counseling (ODC) groups. The mean difference was 0.80 (fixed effect: 95%). The CI was 0.69, 0.92, the p-value was <0.0001, and the heterogeneity (I^2^) was 98% (Figure 2).

**Figure 3:**
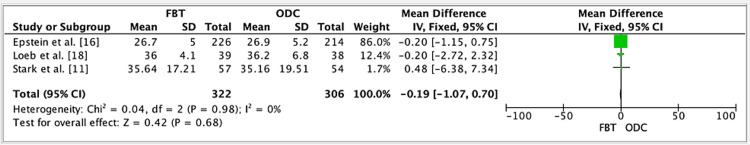
Forest plot for studies about the efficacy of FBT vs. ODC groups References: [11,16,18] CI: confidence interval; FBT: family-based treatment; ODC: occasional diet counseling; SD: standard deviation

The results of three studies showed an odd ratio of -0.19 in the efficacy of FBT vs. ODC groups (fixed effect: 95%). The CI was -1.07, 0.70, the p-value was 0.98, and the heterogeneity (I^2^) was 0% (Figure 3).

**Figure 4:**
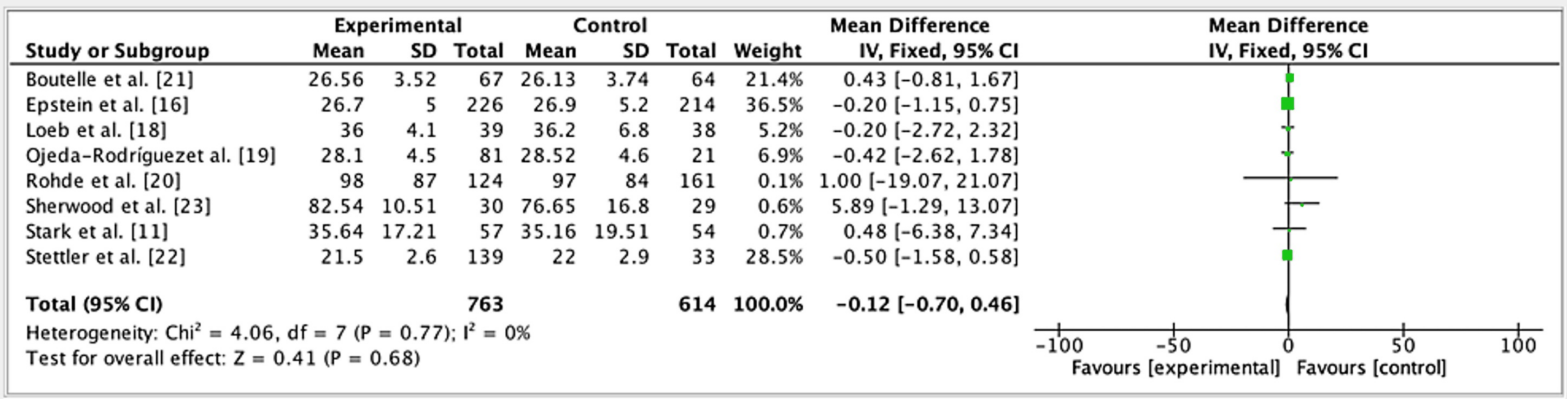
A comparison of the total effectiveness of the FBT and ODC groups* The comparison is best shown by using forest plots References: [11,16,18-23] CI: confidence interval; FBT: family-based treatment; ODC: occasional diet counseling; SD: standard deviation

The results of eight studies were analyzed to compare the effectiveness of FBT against usual care, which was termed ODC. The combined OR was found to be -0.12, with a 95% CI ranging from -0.70 to 0.46. This indicates that the difference between FBT and ODC was not statistically significant (p=0.77), suggesting that FBT did not show a substantial advantage over ODC in these studies. Importantly, the heterogeneity among the studies was 0%, meaning the results were consistent across the different studies analyzed. Figure 4 provides a detailed visualization, illustrating the average effects and standard deviations of each study, highlighting the comparative impact of FBT on reducing pediatric obesity versus the control group using ODC.

**Figure 5:**
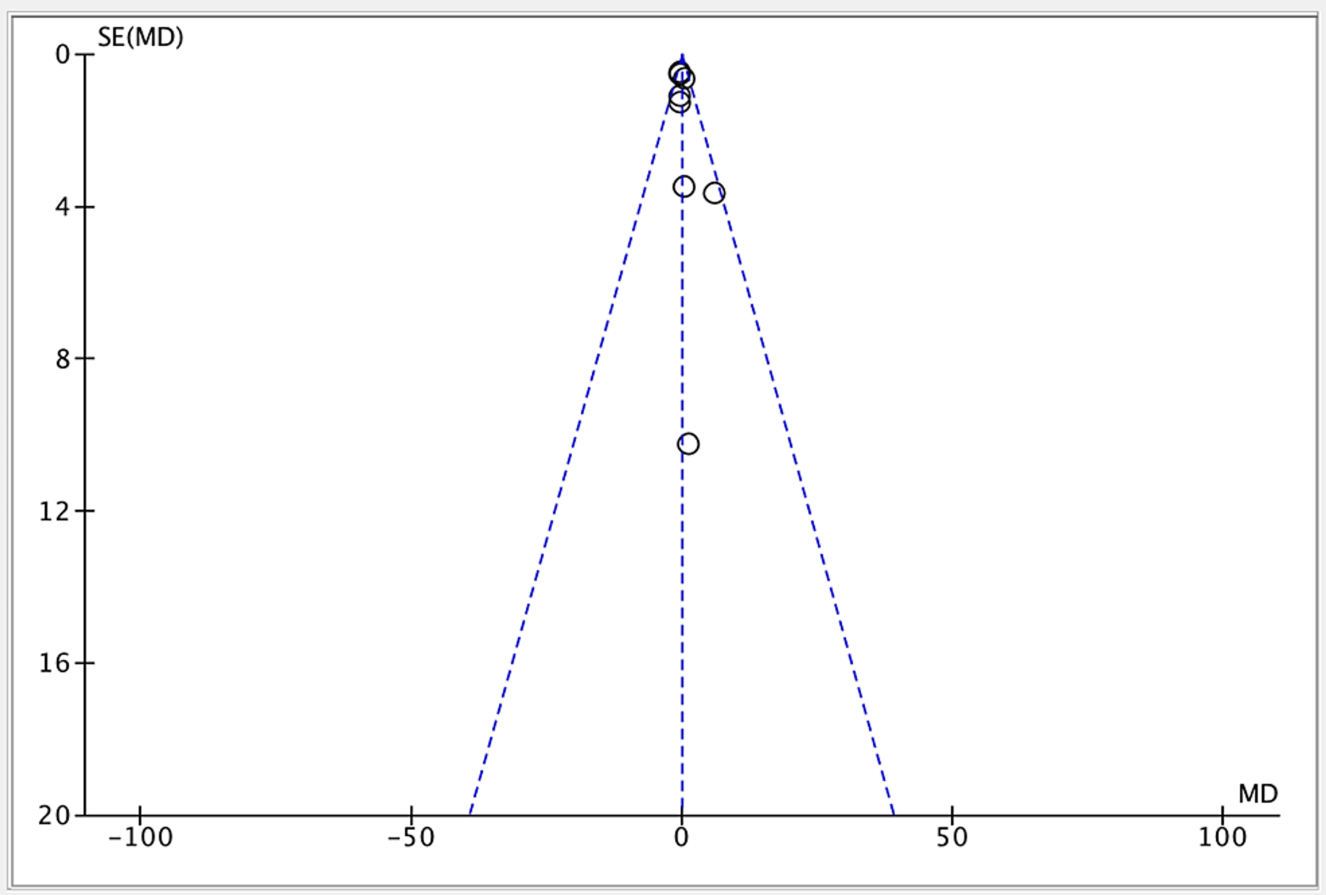
Funnel plot for all included studies about the efficacy of FBT vs. ODC groups References: [11,16,18-23] FBT: family-based treatment; ODC: occasional diet counseling MD: mean difference; SE: standard error

Publication bias was seen in one of the eight studies, as illustrated in Figure 5.

Discussion

Childhood obesity often progresses into adulthood. In a significant longitudinal study, it was found that 84% of children with obesity continue to be obese in adulthood, with this figure rising to 100% among those with severe obesity [17]. These findings underscore the necessity for innovative and evidence-based strategies to manage obesity effectively, which will not only improve health outcomes but also reduce associated costs and enhance family well-being. To this end, we reviewed 10 articles that explored the efficacy of FBT strategies, including in-person and telephonic coaching by health professionals, along with diet, physical activity, and nutritional education for children.

Epstein et al. conducted an RCT that demonstrated the success of family-based behavioral treatments for childhood obesity within pediatric primary care. This intervention yielded better weight management outcomes for the children involved, their parents, and their siblings compared to usual care [16]. This approach shows potential for families with multiple children experiencing obesity. Kinlin et al. highlighted the feasibility of implementing obesity interventions in primary care settings, given that children typically have several primary care visits in their early years. They cautioned, however, against premature expansion of tertiary care-public health partnerships without proven effectiveness, due to the substantial resources such expansions would entail [17]. The study also identified a lack of adequate information provided to parents at the point of referral, which impacted family participation and expectations, a critical factor in the continuity of engagement in pediatric weight management programs [17].

Pbert et al.'s RCT evaluated the effectiveness of telephonic coaching facilitated by a centrally located service to assist families in adopting lifestyle changes recommended by the American Academy of Pediatrics. The "Fitline" program, if successful, could set new standards for pediatric weight management [13]. Stark et al.'s clinic-based behavioral intervention successfully reduced weight in preschoolers, although sustaining these results post-treatment proved challenging [11]. This underscores the need for a consistent outcome measure that accurately reflects changes in adiposity, especially in younger children, where standard measures like the BMI z-score may be less effective due to ceiling effects [11].

Loeb et al.'s RCT on family-based pediatric obesity treatment did not significantly reduce BMI z-scores but helped stabilize the weight gain trajectory in adolescents, aligning with literature that supports parental involvement in managing pediatric obesity [18]. According to Ojeda Rodriguez et al., an intensive lifestyle intervention for children and adolescents with abdominal obesity led to improvements in BMI standard deviation scores and dietary habits, as evidenced by higher diet quality scores [19]. Rodhe et al.'s study targeted obesity-prone young children using a primary prevention intervention, which successfully reduced total energy intake without altering the overall macronutrient and food intake composition. This reduction was primarily in carbohydrates and added sugars, suggesting potential benefits in preventing excessive weight gain [20].

Boutelle et al. compared parent-based treatment (PBT) with family-based weight loss treatments, finding similar outcomes in changes in child and parent weights and lifestyle behaviors. PBT provides the flexibility of scheduling around only the parent’s availability and emphasizes the parent's role as the primary change agent, enhancing parental self-efficacy in managing their child’s weight and other behaviors [21]. Stettler et al.'s RCT emphasized the role of pediatric primary care providers in obesity prevention, suggesting the necessity of training, continuous support, and appropriate compensation for clinicians to effectively implement these interventions [22]. This approach has been shown to significantly impact weight-related outcomes in children from motivated families.

Sherwood et al.'s findings from their RCT support the feasibility and potential effectiveness of pediatric primary care-based interventions in promoting healthy growth among preschool-aged children who are already overweight. Their study, "Healthy Homes/Healthy Kids Preschool," suggests future directions for enhancing obesity prevention strategies, including engaging parents more effectively, supporting pediatric care providers, and refining the timing and modality of intervention delivery based on individual child and family characteristics [23].

Our systematic review underscores the urgent need for effective family-based interventions to address pediatric obesity, highlighting various successful models and identifying areas for future research and application. By synthesizing the findings from diverse studies, we aim to provide a well-rounded view of current methodologies and their outcomes, facilitating better-informed decisions in healthcare strategies for managing childhood obesity.

Limitations

While conducting this meta-analysis, we encountered a diverse range of sample sizes across the included primary studies. The variation in sample sizes presented certain challenges in ensuring consistent statistical power and generalizability. Smaller studies may have contributed to increased variability, while larger studies provided more robust data. This heterogeneity necessitated careful weighting and sensitivity analysis to accurately synthesize the results. Despite these limitations, the meta-analysis offers valuable insights into the studied medical topic.

## Conclusions

The persistence of childhood obesity into adulthood underscores the pressing need for innovative and evidence-based management strategies. Our review of 10 articles highlights the potential of FBT strategies, including behavioral interventions and telephonic coaching, to significantly improve weight management outcomes. Successful models, as discussed, demonstrate the feasibility and effectiveness of integrating these interventions into pediatric primary care. However, sustaining long-term outcomes remains a challenge, necessitating consistent measures to accurately track changes in body fat. Additionally, the crucial role of parental involvement and pediatric primary care providers calls for comprehensive approaches that engage families and support healthcare professionals. Our systematic review offers a well-rounded perspective on current methodologies, paving the way for more informed and effective healthcare strategies to manage and prevent childhood obesity.
